# Frequency of Maternal Anemia in Patients Presenting With Preterm Premature Rupture of Membranes

**DOI:** 10.7759/cureus.52973

**Published:** 2024-01-26

**Authors:** Naila Khan, Saima Khattak

**Affiliations:** 1 Obstetrics and Gynaecology, Medical Teaching Institution, Lady Reading Hospital, Peshawar, PAK

**Keywords:** fetal, anaemia, maternal, premature rupture, preterm

## Abstract

Background: Preterm premature rupture of membranes (PPROM) is the rupture of fetal membranes before 38 weeks of gestation. The etiology is multifactorial. Maternal anemia is one of the factors leading to PPROM. This study aims to determine the frequency of maternal anemia in patients presenting with PPROM.

Materials and Methods: This cross-sectional study was carried out at the Department of Obstetrics and Gynecology, Lady Reading Hospital, Peshawar. This study was conducted from July 1 to December 31, 2021. One hundred and twenty two patients with PPROM presenting to the Department of Obstetrics and Gynecology were included. The diagnosis of PPROM was made based on the history of the PV leak, followed by confirmation with the nitrazine litmus test, microscopic fern test, and ultrasonographic amniotic fluid index measurement. Anemia was determined by examination of hemoglobin levels in the maternal blood samples. Hb <11gm/dl was labeled as anemia. IBM Corp. Released 2011. IBM SPSS Statistics for Windows, Version 20.0. Armonk, NY: IBM Corp. was used for statistical analysis.

Results: The age of the patients ranged from 20 to 40 years. The mean age was 29.14 ± 6.194 years. 63 patients (51.6%) were multiparous (parity 2 to 4). Maternal anemia was observed in 39 patients (32.0%). A significant association (p = 0.005) was observed between maternal anemia and grand multiparity (parity 2 to 4).

Conclusion: Maternal anemia significantly contributes to PPROM, especially in multiple pregnancies. Meticulous family planning and consistent obstetrical monitoring throughout pregnancy are key to addressing this, potentially reducing both maternal anemia risk and PPROM complications.

## Introduction

Pregnant women's anemia is a major global health problem that influences both the development of the fetus and the health of the mother [1]. Preterm delivery is one of the major challenges of pregnancy and is often related to circumstances about the health of the mother [2]. Maternal anemia and preterm deliveries have been linked; this association has been studied extensively since it has significant consequences for prenatal care and newborn outcomes [3].

However, the exact incidence of maternal anemia in women who give birth prematurely still requires further investigation [4]. Fetal membranes, which are made up of amnion and chorion, are a layer of strong membranes that surround the fetus in the mother's uterus throughout gestation. The health of the fetus depends on the integrity of these membranes. Although the rupture of these membranes usually happens at 37 weeks of gestation, it may nevertheless occur before then, with various consequences for both the mother and the fetus [5].

Irfan et al., from Abbottabad, reported that maternal anemia is associated with low birth weight and also with increased admission to the NICU. Shahid et al. from Lahore revealed that 88.3% of pregnant women were not reported to have a PROM history, whereas anemia was noted in 10.6% of pregnant women. Sultana et al., from Karachi, reported a PROM frequency of 3.27%. PPROM prevalence is 3.1% in Brazil, 2.2% in India, 5.3% in Egypt, and 7.5% in Uganda, according to the reported prevalence rates for PPROM. 3% to 4.5% of pregnancies become complicated due to PPROM [6]. Preterm delivery, neonatal sepsis, intrauterine growth retardation, and intrauterine fetal death are the fetal problems associated with PPROM. Placental abruption, postpartum hemorrhage, and chorio-decidual infection are associated with maternal complications [7, 8]. Maternal anemia is hypothesized to cause PPROM by weakening the immune system and making the body more susceptible to infections, while the precise process is unknown [9]. Maternal anemia was seen in 28.3% of women with PPROM in research by Pratiwi et al. 34.75% of patients presenting with PPROM were found to have maternal anemia in research conducted by Kumari et al. [3].

The precise mechanism behind the occurrence of PPROM in the context of maternal anemia remains poorly understood. Anemia is hypothesized to induce changes in the regulation of immune cells, such as T cells and B cells [10]. Consequently, their ability to multiply, engulf particles, and kill bacteria may be diminished. Infection in the amniotic fluid and membranes raises the likelihood of PROM and preterm birth by triggering the release of additional cytokines. Furthermore, these cytokines increase susceptibility to membrane rupture [11]. Several variables contribute to the complexity of preterm delivery, and the most important is the mother's health. Given its possible impact on the health of the fetus, maternal anemia should be thoroughly studied during preterm deliveries. The results seek to clarify any possible links between anemia and preterm birth rates, in addition to measuring the prevalence of anemia in this group of pregnant moms. By better understanding the incidence of maternal anemia in this community, this study hopes to pave the path for improved prenatal care measures that will reduce the risk factors linked to premature births. Lady Reading Hospital is the largest hospital in the province of Khyber Pakhtunkhwa, Pakistan. The majority of serious cases, including maternal and other types of emergency cases, are entertained at Lady Reading Hospital. It is equipped with the latest technologies and has highly trained and well-proficient staff available. Therefore, most patients are visiting there and were selected to conduct this important project. This study aimed to determine the prevalence of maternal anemia in a group of patients who had premature deliveries.

## Materials and methods

This cross-sectional study was carried out from July 1st, 2021, to December 31st, 2021. The study was performed in the Department of Obstetrics and Gynecology, Lady Reading Hospital, Peshawar, Pakistan. The sample size, considering an 8% of margin of error and a 95% of confidence level, was n=122, as computed by the World Health Organization (WHO) calculation based on an anticipated frequency of maternal anemia (p=28.3%). To choose participants, non-probability sequential sampling was used.

According to the operational criteria, women from ages 20 to 40 who had singleton pregnancies between 28- and 36-weeks gestational age and parity from 1 to 4 and were diagnosed with PPROM were included in the research. Less than 20 years of age for marriage is considered to be a risk factor for pregnancy. Therefore, cases younger than 20 years were not selected. Moreover, the gestational age between 28 and 36 weeks was considered an important period of pregnancy. Patients having recent blood transfusions, a history of cervical operations or caesarean births, blood dyscrasias, connective tissue abnormalities, traumatic fetal membrane ruptures, or uterine ruptures were among the exclusion criteria. Moreover, multiple pregnant mothers and mothers with several gestational diseases, including hypertension, diabetes mellitus, and renal disorders, were also excluded from the study. Strict adherence to these exclusion standards was intended to reduce prejudice.

After receiving clearance from the study department and ethics committee, 122 patients from the obstetrics and gynecology departments at Lady Reading Hospital were enrolled. The ethical approval was obtained from the hospital ethical review board with reference number 117/LRH/MTI. Informed consent was obtained from each patient before conducting the study, and the aim of this study and other details were communicated verbally and in writing to all participants.

Clinical parameters and demographic data were documented, including height, weight (kg), parity, age (years), BMI (kg/m2), gestational age (weeks), and duration of complaints in days. Obesity criteria are defined as per CDC criteria. At the time of admission, a complete physical examination of participants that included a sterile speculum, abdominopelvic, and general examination was conducted after a detailed medical and obstetrical history was obtained.

For the determination of anemia, 3ml of EDTA tubes were labeled as per the patient ID, and whole blood was collected using the disposal syringe of 5ml after rubbing the alcohol swab before inserting the needle. Blood was collected in EDTA tubes (Xenle Company, China). The EDTA tube was put on the hemotology shaker/ mixer in order to mix the blood with the EDTA reagent in the tube. After mixing the blood, the whole blood sample was determined through the Sysmex KX21 Hematology Analyzer (Japan). The blood samples were taken to determine the hemoglobin level. The frequency of maternal anemia, defined as a hemoglobin level below 11 gm/dl-was documented during the third and first trimesters, while during the second trimester, less than 10.5 g/dl were considered anemia. 

All the collected data and information related to patients were initially entered in the Microsoft Excel Sheet 2020. Then, the collected data were properly coded, and inserted, and analyzed in IBM Corp. Released 2011. IBM SPSS Statistics for Windows, Version 20.0. Armonk, NY: IBM Corp. The frequencies, along with percentages, were calculated for the quantitative data. The mean and standard deviation (±SD) were calculated for weight, parity, age, gestational age, and BMI. Percentages and frequencies were arranged in tables for better representation. Qualitative data were also shown as numbers and percentages. Age, gestational age, BMI, length of complaints, and parity were among the impact modifiers that were adjusted for by stratification. A post-stratification chi-square test was used for inferential analyses of variables. The level of significance was also determined, with P ≤0.05 being considered statistically significant; otherwise, the data were considered non-significant. 

## Results

Important baseline and demographic information about the research participants are summarized in Table 1. It provides a thorough overview of important metrics, providing important details on the characteristics of the cohort. With a standard deviation of 6.194, the participants' average age was 29.14 years, indicating a diversity of ages within the sample. The subjects' average gestational age was 33.97 weeks. The participants’ average body mass index (BMI) was 23.295 kg/m2. A low standard deviation of 0.212 indicates that the participants' reported problems were generally constant, with an average duration of 09.03 days.

**Table 1:** Demographic and baseline characteristics of the participants.

Demographics & Baseline characteristics	Mean ± std. deviation
Patient Age (years)	29.14 ± 6.194
Gestational Age (weeks)	33.97 ± 0.999
BMI (kg/m^2^)	23.295 ± 2.6808
Patient Weight (kg)	58.99 ± 6.068
Duration of Complaints (days)	09.03 ± 0.212

Table 2 shows frequencies and percentages for each category and provides an extensive analysis of the participant demographics. Of the participants, 45.1% were between the ages of 20 and 30, and 54.9% were between the ages of 31 and 40. 40.2% of gestational-age cases fell between 28 and 32 weeks, while 59.8% of cases fell between 33 and 36 weeks. The distribution of BMI categories showed that 30.3% of people were classified as "overweight" (25.0-29.9 kg/m2), 11.5% as "obese," and 58.2% as "healthy" (8.5-24.9 kg/m2). In terms of parity, 51.6% had paras 2-4 and 48.4% were primiparous (para 1). Regarding the length of complaints, 35.2% of complaints were longer than 10 days, while 64.8% of complaints were less than 10 days. Regarding maternal anemia, 68% of participants were classified as having no maternal anemia, whereas 32% of individuals were classified as having maternal anemia.

**Table 2:** Cross-tabulation of the variables included in the study.

Variables	Detail	Frequency	Percentage
Age Group	20 to 30 years	55	45.1
	31 to 40 years	67	54.9
Gestational Age	28 to 32 weeks	49	40.2
	33 to 36 weeks	73	59.8
BMI Status (kg/m^2^)	Healthy (8.5-24.9)	71	58.2
	Overweight (25.0–29.9)	37	30.3
	Obese (30.0 and above)	14	11.5
Parity	Para 1	59	48.4
	Para 2-4	63	51.6
Duration of complaints (days)	10 days or below	79	64.8
	More than 10 days	43	35.2
Maternal anemia	YES	39	32.0
	NO	83	68.0

Table 3 presents frequencies, percentages, and corresponding p-values for each variable and offers a thorough overview of the association between maternal anemia and various important factors examined. Of the individuals in the 20 to 30 age range, 29.1% had maternal anemia. 34.3% of mothers in the 31 to 40 age range had maternal anemia, while 64.7% were unaffected. There is no significant correlation between age and maternal anemia (p-value of 0.537). In terms of BMI classifications, "healthy" (29.6%), "overweight" (35.1%), and "obese" (35.7%) are associated with maternal anemia. The corresponding p-value was 0.799, indicating that there was no significant correlation between maternal anemia and BMI. Regarding gestational age, the group 28 to 32 weeks showed 28.6% of maternal anemia, and the group 33 to 36 weeks showed 34.2% of maternal anemia, with no significant correlation between gestational age and maternal anemia (p=0.509). There is a significant correlation between parity and maternal anemia (p=0.007), as shown in Table 3.

**Table 3:** Association of maternal anemia with the included parameters.

Variable	Detail	Maternal Anemia		P-Value
		Yes	No	p-value = 0.537
Age (Years)	20 to 30	16 (29.1%)	39 (70.9%)	
	31 to 40	23 (34.3%)	44 (64.7%)	
BMI (kg/m2)	Healthy	21 (29.6%)	50 (70.4%)	p-value = 0.799
	Overweight	13 (35.1%)	24 (64.9%)	
	Obese	5 (35.7%)	09 (64.3%)	
Gestational Age	28 to 32 weeks	14 (28.6%)	35 (71.4%)	p-value = 0.509
	33 to 36 weeks	25 (34.2%)	48 (65.8%)	
Duration of Complaints	10 days or below	31 (39.2%)	48 (60.8%)	p-value = 0.923
	More than 10 days	08 (18.6%)	13 (81.4%)	
Parity	1	12 (20.3%)	47 (79.7%)	p-value = 0.007
	2 to 4	27 (42.9%)	36 (57.1%)	

## Discussion

Pregnant individuals are susceptible to anemia. First, hemodilution occurs during pregnancy, which is a physiological process. However, it may also become pathological when there is an imbalance in the metabolism of certain micro- and macronutrients. Maternal anemia has several harmful consequences for both the mother and the baby, and one such impact is the occurrence of PPROM [10].

It is still unclear exactly what mechanism causes PPROM to arise in the setting of maternal anemia. It is theorized that anemia causes modifications in the way immune cells like T and B cells are regulated. As a result, they could be less able to grow, take up particles, and destroy germs. By inducing the production of extra cytokines, infection in the amniotic fluid and membranes increases the risk of PROM and premature delivery. Moreover, these cytokines make membrane rupture more likely [11].

Maternal employment is an additional risk factor for PPROM. The workplace environment and maternal stress coexist with anemia. Stress induces the secretion of adrenaline and norepinephrine. Iron deficiency anemia also results in heightened secretion of these stress hormones. Anxiety and stress may lead to the activation of corticotropin-releasing hormone (CRH). The synergistic impact of heightened levels of adrenaline, norepinephrine, and CRH results in prenatal hypertension, pre-eclampsia, preterm birth, and PROM. Hypoxia at the tissue and cellular level is also a consequence of the low hemoglobin level. This occurrence leads to cellular harm caused by free radicals, ultimately raising the risk of PPROM [12].

Maternal anemia was detected in 39 patients (32.0%) who had PPROM in the current research. The average age of the patients in this research was 29.14 ± 6.194 years. In contrast to this research, another study reported a slightly higher mean mother age of 31.4 ± 5.6 years [13]. The decrease in the average age seen in this research may be attributed to the prevalence of Islam in our community since Muslims prefer to marry at a younger age in comparison to Western societies.

The age group of 31 to 40 years accounted for the highest number of patients (67, 54.9%) with PPROM. Although this research did not find a statistically significant correlation between maternal anemia and age (p = 0.537) in patients with PPROM, most patients (23 out of 39 patients, 59%) with anemia were between the ages of 31 and 40. The age of females has a direct correlation with their reproductive function. As individuals get older, the functionality of their reproductive organs declines. These impaired functions may hinder the fertility, quality, and number of eggs and embryogenesis. Hence, symmetrical and asymmetrical intrauterine fetal development limits, congenital anomalies, PPROM, and preterm delivery may manifest throughout pregnancy [13-15]. This research observed an increase in the occurrence of PPROM during early pregnancy, specifically with higher frequencies. Pregnancies that occur throughout adolescence are also prone to causing maternal and fetal difficulties. In this age range, the underdeveloped reproductive organs are not capable of sustaining a pregnancy. The amniotic membranes have a lower number of immune cells and are more prone to rupture, leading to PPROM [16].

Having many pregnancies (i.e., two or more) is the most influential factor that increases the chances of maternal anemia in the context of PPROM. The probability is significantly increased if the patient has a negative obstetrical history with both surgical and vaginal methods of birth [17]. The study conducted by Caughey et al. demonstrates the importance of maternal anemia in the context of PPROM. The research reveals that multiparous women are three times more likely to experience maternal anemia compared to nulliparous women [18].

The present investigation has a few limitations, such as being a single-center study conducted in a single-district hospital. Moreover, the sample size was also smaller. Furthermore, nutritional components were not determined, which is also an important confounding factor in anemia, particularly in pregnant females.

## Conclusions

Research findings suggest that maternal anemia is a significant contributing factor to PPROM. A robust and noteworthy correlation has been identified between multiple pregnancies and the occurrence of maternal anemia. To mitigate this issue effectively, proactive measures involving meticulous family planning practices and consistent adherence to obstetrical monitoring during the entire gestational period are strongly recommended. These strategies aim to address the risk associated with maternal anemia, potentially reducing the incidence of PPROM and its related complications during pregnancy. It is important for implementation to focus on pregnant females, particularly in the second and third trimesters, where iron requirements increase with the growth of the fetus. It is also crucial to initiate an awareness campaign for such an underdeveloped area where basic requirements are not sufficient.
